# Perilipin1 Deficiency in Whole Body or Bone Marrow-Derived Cells Attenuates Lesions in Atherosclerosis-Prone Mice

**DOI:** 10.1371/journal.pone.0123738

**Published:** 2015-04-09

**Authors:** Xiaojing Zhao, Mingming Gao, Jinhan He, Liangqiang Zou, Ying Lyu, Ling Zhang, Bin Geng, George Liu, Guoheng Xu

**Affiliations:** 1 Department of Physiology and Pathophysiology, School of Basic Medical Sciences, Peking University, Beijing, China; 2 The Key Laboratory of Molecular Cardiovascular Sciences, the Ministry of Education, Beijing, China; 3 Department of Pharmacy, State Key Laboratory of Biotherapy, West China Hospital of Sichuan University, Chengdu, Sichuan, China; University of Munich, GERMANY

## Abstract

**Aims:**

The objective of this study is to determine the role of perilipin 1 (Plin1) in whole body or bone marrow-derived cells on atherogenesis.

**Methods and Results:**

Accumulated evidence have indicated the role of Plin1 in atherosclerosis, however, these findings are controversial. In this study, we showed that Plin1 was assembled and colocalized with CD68 in macrophages in atherosclerotic plaques of ApoE-/- mice. We further found 39% reduction of plaque size in the aortic roots of Plin1 and ApoE double knockout (Plin1-/-ApoE-/-) females compared with ApoE-/- female littermates. In order to verify whether this reduction was macrophage-specific, the bone marrow cells from wild-type or Plin1 deficient mice (Plin1-/-) were transplanted into LDL receptor deficient mice (LDLR-/-). Mice receiving Plin1-/- bone marrow cells showed also 49% reduction in aortic atherosclerotic lesions compared with LDLR-/- mice received wild-type bone marrow cells. In vitro experiments showed that Plin1-/- macrophages had decreased protein expression of CD36 translocase and an enhanced cholesterol ester hydrolysis upon aggregated-LDL loading, with unaltered expression of many other regulators of cholesterol metabolism, such as cellular lipases, and Plin2 and 3. Given the fundamental role of Plin1 in protecting LD lipids from lipase hydrolysis, it is reasonably speculated that the assembly of Plin1 in microphages might function to reduce lipolysis and hence increase lipid retention in ApoE-/- plaques, but this pro-atherosclerotic property would be abrogated on inactivation of Plin1.

**Conclusion:**

Plin1 deficiency in bone marrow-derived cells may be responsible for reduced atherosclerotic lesions in the mice.

## Introduction

All eukaryotic cells are able to store lipids in cytosolic lipid droplets (LD) [1,2]. The cellular lipid storage in non-adipose tissue is important with regard to the development of various abnormalities, such as steatosis and atherosclerosis (As). In As, macrophages take up lipids in an uncontrolled manner making them transform into foam cells, the hallmark of As[3].

Plin1(perilipin)/Plin2(adipophilin/ADRP)/Plin3(Tip47)(PAT family) were found in macrophages and foam cells [4–6]. In adipocytes, they were distributed in the surface of the LDs of different sizes. Plin1 was found in high abundance in adipocytes and steroidogenic cells [7,8], whereas, Plin2 (ADRP) is expressed ubiquitously [9]. Plin1 located on the surface of LDs to protect them from lipolysis. This protective effect disappears when Plin1 is phosphorylated by PKA and translocated to cytoplasm [10,11].

The role of Plin1 in atherogenesis had been implicated when Plin1 was found up-regulated in foam cells in human atherosclerotic plaques[12,13]. Expression of Plin1 is gradually increased in the differentiation of monocytes into macrophages[4]. Transfection of Plin1 in Chinese Hamster Ovary cells, human hepatoma cell line HepG2 cells and rat McArH7777 cells resulted in lipid droplet formation[14]. Moreover, overexpression of Plin1 in macrophage line THP-1 cell could increase lipid accumulation[4], transforming the cells to foam cell-like macrophages. By contrast, Langlois et al. reported that Plin1 deletion in male LDLR-/- mice showed accelerated As, with concurrent upregulation of ABCA1 and ABCG1[15]. Thus, the role of Plin1 in As and lipid metabolism is still uncertain. It is therefore necessary to investigate this issue further to clarify the role(s) of Plin1 in As and explore the potential mechanism involved in the process.

In our approach, we first demonstrated the presence of Plin1 in macrophages in atherosclerotic plaque of ApoE-/- mice by immunofluorescence staining. To investigate the role of Plin1 in As, we generated Plin1 and ApoE double knockout (Plin1-/-ApoE-/-) mice. Quantification of As in aortic roots indicated 39% decrease of As in Plin1-/-ApoE-/- mice compared with ApoE-/- littermates. To further clarify the nature of Plin1 in As, bone marrow cells (BMCs) with or without Plin1 were transplanted to irradiated LDLR-/- recipients. LDLR-/- mice that received BMCs without Plin1 showed attenuated As progression compared with that of BMCs with Plin1. When macrophages isolated from Plin1-/- mice were incubated with acetylated LDL or aggregated LDL in vitro, they showed reduced lipid accumulation, especially cholesterol ester inside the cells.

## Materials and Methods

### Antibodies

Polyclonal antibodies against Plin1 or Plin2 [7, 8, 10, 16] were gifts from the laboratory of C. Londos (US National Institutes of Health). This Plin1 antibody was used for immunostaining and another anti-Plin1 antiserum from Abcam (#ab3526) was used for immunoblotting. The sources of other antibodies used were listed as follows: rabbit anti-ATGL from Cayman Chemical (Cat# 10006409), rabbit anti-HSL from Cell Signaling Technology (Cat# 4107s), rat anti-CD36 from (R&D, MAB1955), mouse anti-ABCA1 (Abcam, ab18180), and mouse monoclonal anti-CD68 (Abcam, #ab955),

### Animals and animal experiments

All animal experiments were conducted following protocols approved by the Institutional Animal Care and Use Committee at Peking University Health Science Center (No.La2010-061). For surgery, mice were anesthetized with pentobarbital sodium (50 mg/kg, i.p.) and local lidocaine infiltration. Plin1-/- mice on a 129/SvEv background were from the laboratory of C. Londos (US National Institutes of Health) [17,18]. This Plin1-/- mouse strain were crossed with the ApoE-/- mouse in C57BL/6J background to generate the F1 offspring (Plin1-/-ApoE-/-) with double ablation of the Plin1 and ApoE loci. The Plin1-/-ApoE-/- mice were continuously backcrossed with ApoE-/- mice 6 times to obtain the F6 generation of Plin1-/-ApoE-/- mice on a pure C57BL/6 background. The ApoE-/- littermates were used as control animals. All mice were housed and bred in a pathogen-free barrier facility.

At 8 weeks of age, 14 males and 20 females of Plin1**-/-**ApoE**-/-** mice, and 14 males and 22 females of ApoE**-/-** mice were fed a high fat Western diet (21% fat, 0.15% cholesterol, 19.5% casein, no sodium cholate; w/w) for 12 weeks. Atherosclerotic lesion was then analyzed in both genotypes of the mice. For bone marrow transplant study, LDLR**-/-** females with similar plasma cholesterol levels at 8 weeks old were distributed into 2 groups (n = 10 in each group) and subjected to a 9-Gy total body irradiation by using a cesium gamma source. Bone marrow cells used for repopulation were extracted from the femur and tibia of Plin1**-/-** or Plin1+/+ male mice at 6 weeks old. Six hours after irradiation, 5×10^6^ bone marrow cells from either Plin1**-/-** or Plin1+/+ male mice were suspended in 300 μl of RPMI-1640 medium and injected in irradiated mice via the tail vein. These mice were fed with similar Western diet for 12 weeks, and then atherosclerotic lesion development was analyzed.

Plasma levels of cholesterol (TC) and triglyceride (TG) were measured by using of the enzyme-coupled assay kits (Applygen Technologies, Beijing)

### Histology

Atherosclerotic lesion area in the aortic root was quantified on cross sections of the aorta as previously described [19]. In brief, mice were sacrificed and flushed with 20 ml phosphate-buffered saline, followed by perfusion of 4% paraformaldehyde through the left ventricle. Series sections of frozen heart tissue at 10 μm thickness were obtained by cutting from the apex of the heart towards the origin of the aorta. Sections were mounted from the point where all three aortic valve cusps became clearly visible. Every fifth section was used for oil red O (ORO) staining and counterstained with hematoxylin, so that each ORO stained slide was separated in tissues with a 50-μm distance. Atherosclerotic lesion areas were measured using NIH image J graphic Analysis System and were reported as the average ORO staining area per section in the first five such sections for each mouse. Lesion area of the aorta en face was quantified using NIH image J software and expressed as percent of the total aortic area or defined regions, such as the aortic arch, thoracic aorta, and abdominal aorta.

### Immunofluorescence staining

The sections of aortic root were blocked with 10% goat non-immune serum, and incubated with the primary antibodies against Plin1 or CD68 for 30 min at room temperature. After washing, different secondary antibodies coupled with TRITC or FITC was added and incubated for 30 min. Images were recorded with using a fluorescence microscope (Leica DMI 3000B, German).

### Preparation of aggregated LDL (aggLDL)

Human LDL (*d =* 1.03 to 1.053 g/ml) were purchased from Peking Union-Biology Co.Ltd. After extensive dialysis against cold PBS to remove KBr, the protein concentration of LDL was adjusted to 1mg/ml by PBS and vortexed vigorously for 1 minute to yield aggLDL[20].

### Experiments in cultured macrophages

Thioglycollate-elicited peritoneal macrophages were harvested as previously described [21]. The peritoneal macrophages were maintained in RPMI 1640 medium containing 25 mmol/L HEPES buffer and 10% fetal calf serum. Cells were seeded in 6-well plates at a density of 2×10^6^ cells/well. In some experiments, 50 μg/ml aggLDL were added to the culture media. After 48 hours incubation with or without aggLDL, cells were washed 3 times with PBS, and then free cholesterol (FC), cholesterol ester (CE) and triglyceride (TG) were determined using commercial kits from Applygen Technologies (Beijing, China).

### Cholesterol efflux assay

The harvested peritoneal macrophages were incubated for 24 h with 5 μg /ml NBD-cholesterol (Invitrogen), a fluorescent analogue of cholesterol, diluted in culture media. NBD-Cholesterol was first dissolved in absolute ethanol (ethanol concentration in cell culture media was under 0.2%). Then the Cells were washed with culture medium, sonicated with a microprobe for 15 seconds, and then centrifuged at 3000rmp/min for 5 min. These wells provided base-line (time 0) values for total NBD-cholesterol content. Other wells of cells were incubated without or with recombinant apoA1 at 10 μg/ml for different time (from 1h to 6h). Cellular cholesterol efflux was quantified by measuring the release of cellular NBD-cholesterol into the medium as a function of time. Two hundred microliter aliquots of the incubation medium were centrifuged for 2 min at 300g to pellet any floating cells. Fluorescence was read with Fluoroskan ascent FL (Labsystems), set at excitation 470 nm and emission 530 nm. The percentage of cholesterol efflux was calculated as a ratio of the fluorescence intensity in the medium against the total fluorescence within cells [22].

### Oil red O and Nile red staining of macrophages

Cells were washed twice with PBS and were fixed with 1 ml 4% formaldehyde for 30 min. Thereafter cells were washed three times with 1 ml PBS and were incubated with 1 ml Oil Red O (0.3% in 60% isopropanol) for 30 min or Nile red (2.5 μg/ml) for 10 min. Cells were again washed twice with PBS. Then the cells were observed under a Nikon Eclipse 50i microscope [23].

### Western blot

Aorta or peritoneal macrophages were isolated from mice and then lysed. The protein content was determined with BCA assay kit (Applygen Technologies, Beijing, China). Equal amounts of proteins were loaded and separated by SDS-PAGE. The proteins transferred on nitrocellulose membranes (Applygen Technologies, Beijing, China) were recognized by primary antibodies and then by secondary antibodies conjugated to horseradish peroxidase. The blots were developed by use of the enhanced chemiluminescence detection reagents. If required, the antibodies bound to membranes were removed by a commercial stripping solution from Applygen Technologies Inc, and then the blots were reprobed with use of other antibodies and developed [24].

### Quantitative real-time PCR

Total RNA was extracted from macrophages and foam cells and first-strand cDNA was generated by using a RT kit (Invitrogen). All samples were quantitated by using the comparative CT method for relative quantitation of gene expression, normalized to GAPDH.

### Statistical analysis

Data are presented as means ± SEM. Statistical comparison between the two groups was performed by Student’s t**-**test and among multiple groups by one-way ANOVA with use of GraphPad Prism 4.0. A value of P <0.05 was considered statistically significant.

## Results

### Plin1 expression in atherosclerotic plaques and its deficiency reduced cholesterol accumulation in peritoneal macrophages

**Fig 1:**
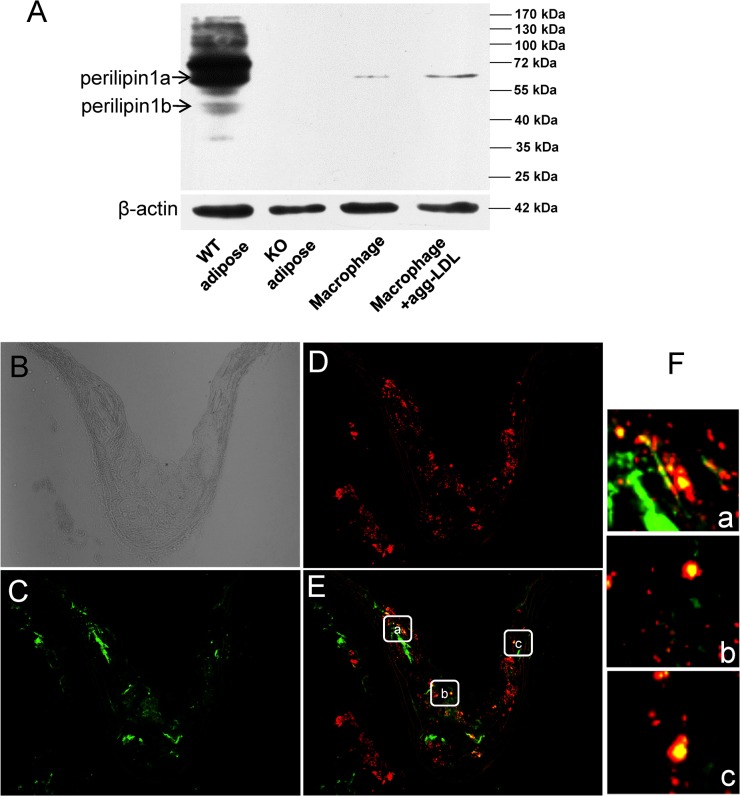
Plin1 expresses in atherosclerotic lesions. (A) Immunoblotting analysis of Plin1 expression in perivascular adipose tissue and in peritoneal macrophages isolated from Plin1-/- and wild-type mice. Incubation with modified LDL increases Plin1 expression in the macrophages. Anti-Plin1 antiserum from (Abcam, #ab3526) recognizes the C-terminal amino-acid residues of Plin1. (B) Unstained frozen section of arotic root of ApoE**-/-** mice. (C) The frozen section of arotic root in ApoE**-/-** mice was immunostained (Green) with the primary antibody against CD68, a macrophagemarker. (D) Immunostaining of Plin1 (Red). (E and F) Merged image shows colocalization of Plin1 and CD68 in atheroma. Boxed area in panel E (a-c) were magnified to show colocalization of Plin1 and CD68 (F, a-c) in macrophages in atherosclerotic lesion in ApoE-/- mice.

**Fig 2:**
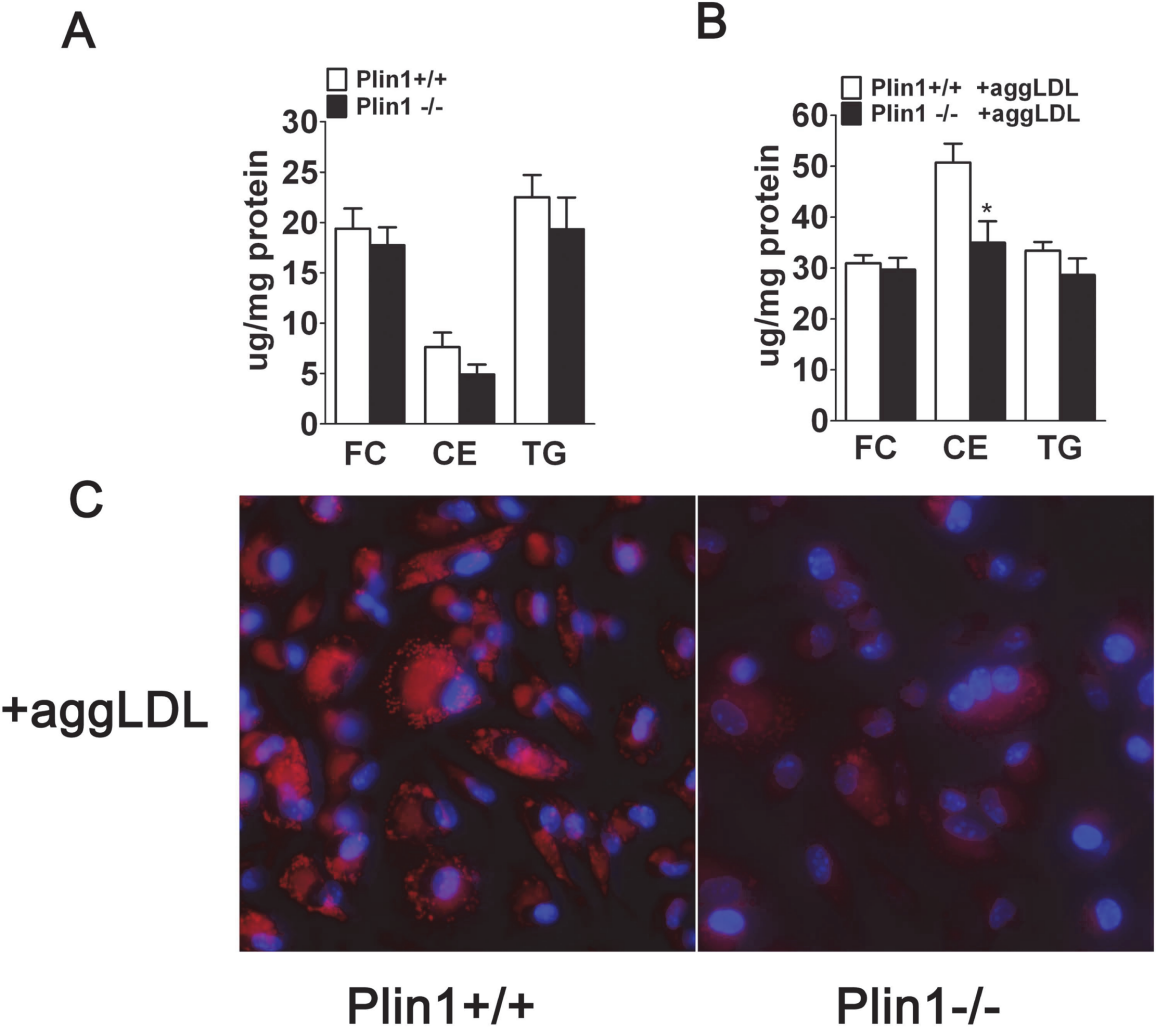
Lipid content is decreased in peritoneal macrophages and foam cells derived from Plin1-/- mice. Peritoneal macrophages from Plin1+/+ and Plin1**-/-** mice were incubated for 48 hours in the absence or presence of 50 μg/ml aggLDL. (A and B) The contents of free cholesterol (FC), cholesterol ester (CE) and triglyceride (TG) were measured. n = 4,*P < 0.05. (C) Nile red staining of LDs in peritoneal macrophages from Plin1+/+ and Plin1**-/-** mice with aggLDL (50μg/ml) (n = 6).

Immunoblotting showed that Plin1 was present in perivascular adipose tissue associated with the aorta (Fig 1A), but was absent in the aortic tissue without the perivascular adipose tissue, in wild-type mice (data not shown). Plin1b, a short variant of Plin1, seemed to express in a trace amount, in the aortic tissue of wild-type mice (Fig 1A). In contrast, the full length of Plin1 protein was detected in peritoneal macrophages of mice and its expression was inducible by incubation with modified LDL (Fig 1A). Immunostaining revealed that Plin1 was expressed in the region of the atherosclerotic plaque in ApoE-/- mice and colocalized with CD68, a marker of the macrophage (Fig 1B–1F). To assess the role of Plin1 in foam cell formation, the peritoneal macrophages isolated from Plin1**-/-** and Plin1+/+ mice were incubated with aggLDL (50 μg/ml) for 48 h. Plin1 deficiency significantly reduced the accumulation of lipid droplets and cholesterol esters (CE) (Fig 2A–2C). Similarly, Plin1 deficiency also attenuated lipid droplet and CE accumulation in macrophages treated with acLDL (data not shown).

### **Reduced atherosclerotic lesions in ApoE**-/- **Mice with Plin1 deficiency**

**Fig 3:**
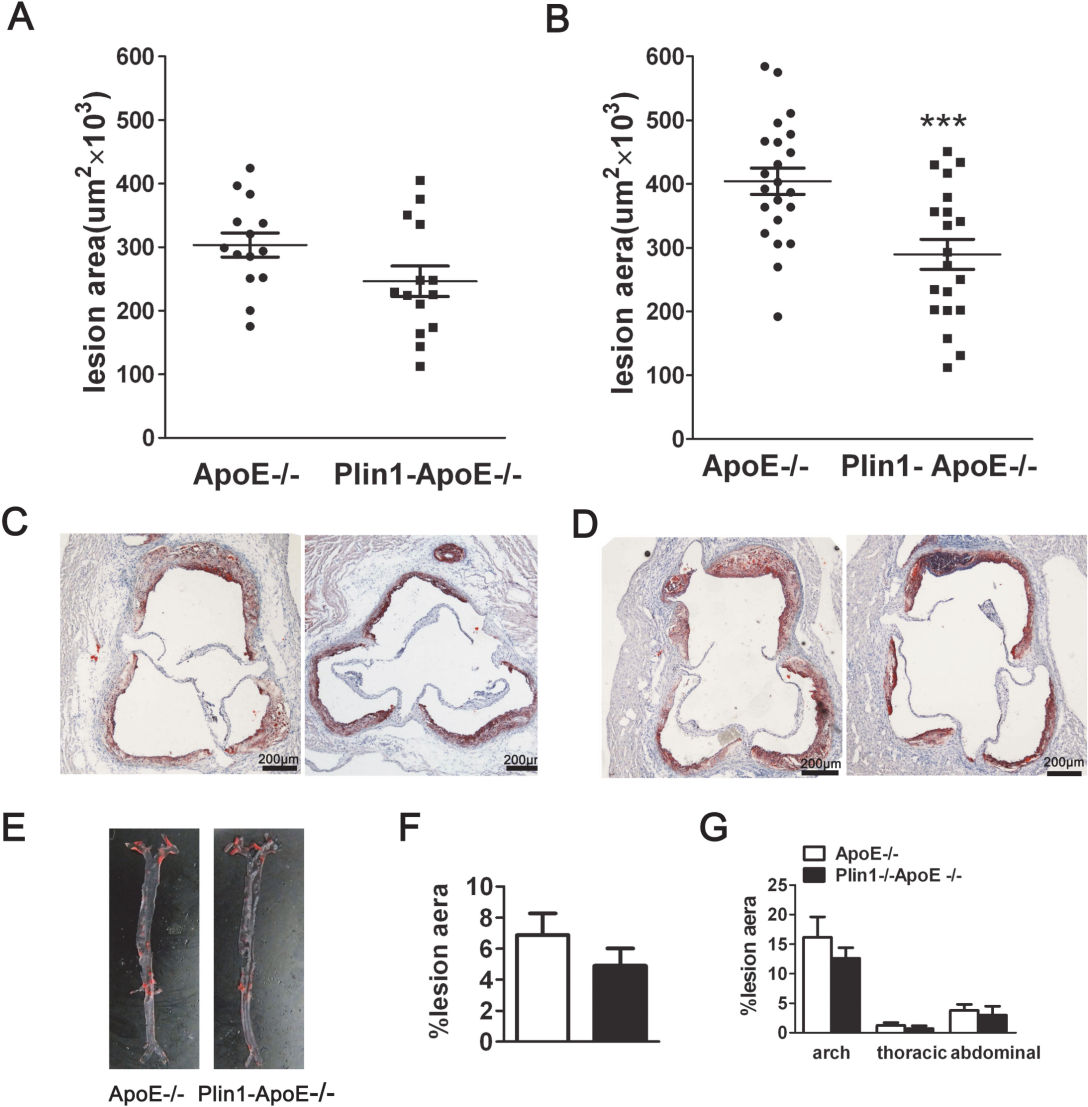
Ablation of Plin1 restricts As in female ApoE-/- mice. (A and B) The mean lesion area (±SEM) in the aortic roots of male (A) and female (B) ApoE*-/-* and Plin1**-/-**ApoE**-/-** mice, *** *p* < 0.001. (C and D) Representative photographs of aortic roots from male (C) and female (D) ApoE**-/-** and Plin1**-/-**ApoE**-/-** mice stained with oil red O. (E) Representative photographs of the aorta en face from female ApoE**-/-** and Plin1**-/-**ApoE**-/-** mice. (F) Quantification of atheroma of total aortic lesion area. n = 15. (G) Regional analysis of lesion distribution in the aorta.

To assess the impact of Plin1 in atherogenesis in vivo, we crossed Plin1**-/-**mice with ApoE**-/-** mice to generate Plin1**-/-**ApoE**-/-** mice and using ApoE**-/-** littermates as controls. Despite the similar levels of plasma cholesterol and triglyceride between ApoE**-/-** and Plin1**-/-**ApoE**-/-** mice, Plin1**-/-**ApoE**-/-** males had reduced body weight compared with ApoE**-/-** male littermates (29.6 ± 0.8 g v.s. 34.2 ± 1.8 g; p = 0.035; n = 20) (Table 1). Quantification of Oil red-stained cross sections of aortic roots did not detect significant difference between male Plin1**-/-**ApoE**-/-** and ApoE**-/-** littermates (Fig 3A and 3C). Unlike males, the body weight of female Plin1**-/-**ApoE**-/-** did not differ from female ApoE**-/-** littermates (Table 1). However, they showed reduced plaque size in aortic roots when compared with female ApoE**-/-** mice (ApoE**-/-** females: 404 ± 21×10^3^ μm^2^ n = 20; Plin1**-/-**ApoE**-/-** females: 290 ± 24×10^3^ μm^2^, n = 22) (Fig 3B and 3D). Histological analysis of aortic root lesions revealed no significant difference in macrophage or smooth muscle cell content in atherosclerotic plaques of Plin1**-/-**ApoE**-/-** mice and ApoE-**/-** mice (data not shown). We also performed en face analysis of the aortic arch and descending aorta in these female mice but the results showed no statistically significant differences in lesion area between the 2 groups of mice, although there was a trend toward decreased lesion area in Plin1**-/-**ApoE**-/-** mice (Fig 3E–3G). Taken together, Plin1 deficiency may have a protective role in As.

**Table 1:** Plasma cholesterol, triglyceride and body weight in ApoE-/- and Plin1-/-ApoE-/-mice after 12 weeks on western diet.

Genotype	n	Body Weight (g)	Total Cholesterol (mg/dL)	Triglyceride (mg/dL)
male				
Plin1-/-ApoE-/-	15	29.59±0.8 *	1464±133.0	107.1±5.7
ApoE-/-	15	34.19±1.8	1371±86.7	103.7±8.6
female				
Plin1-/-ApoE-/-	20	22.78±0.7	1015±89.9	87.9±11.5
ApoE-/-	22	24.61±0.4	1101±99.7	102.1±10.9

*p<0.05 for Plin1**-/-**ApoE**-/-** vs. ApoE**-/-**.

### Role of Plin1-/- bone marrow cells in As

**Fig 4:**
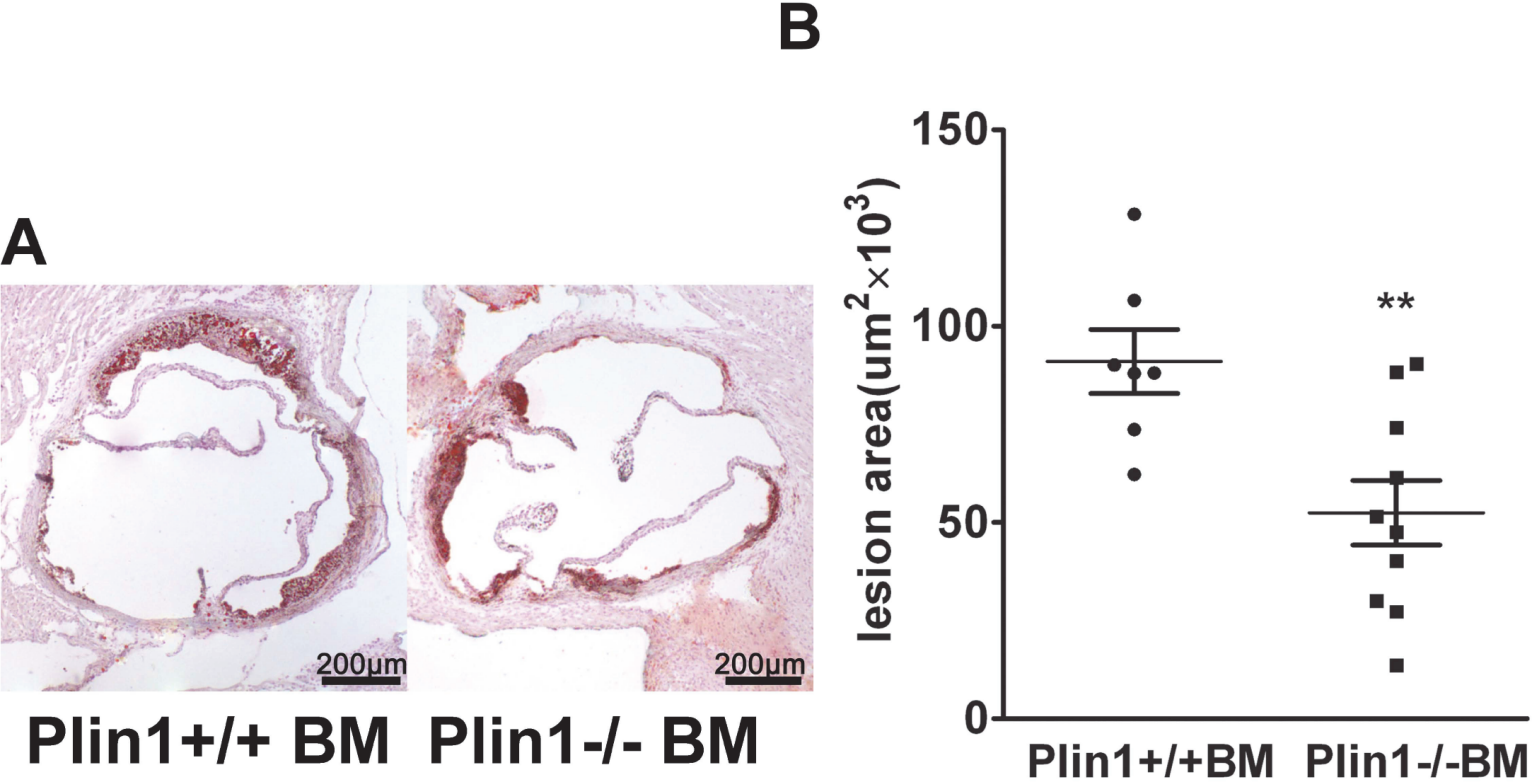
The bone marrow cells from male Plin1-/- mice transplanted to female LDLR-/- mice reduce atherosclerotic lesion. (A) Representative photographs of aortic roots from LDLR**-/-** mice received bone marrow cells from Plin1+/+ and Plin1**-/-** mice respectively. (B) The mean lesion area (±SEM) in the aortic roots in mice transplanted with bone marrow cells from Plin1+/+ or Plin1**-/-** mice. ** *p* < 0.01 for Plin-/- vs. Plin+/+.

To further explore the role of Plin1 in As, bone marrow cells from Plin1+/+ or Plin1**-/-** male mice were transplanted to 8-week-old LDLR**-/-** recipient female mice. Following 12 weeks of Western diet, mice received bone marrow cells from Plin1**-/-** donors had smaller lesions compared with those receiving bone marrow cells from Plin1+/+ donors (from 91 ± 8×10^3^ to 52 ± 8×10^3^ μm^2^) (Fig 4A and 4B). Therefore, absence of Plin1 in bone marrow-derived cells alone is sufficient to reduce As in LDLR**-/-** mice.

### Mechanisms of reduced lipid accumulation in macrophages lacking Plin1

**Fig 5:**
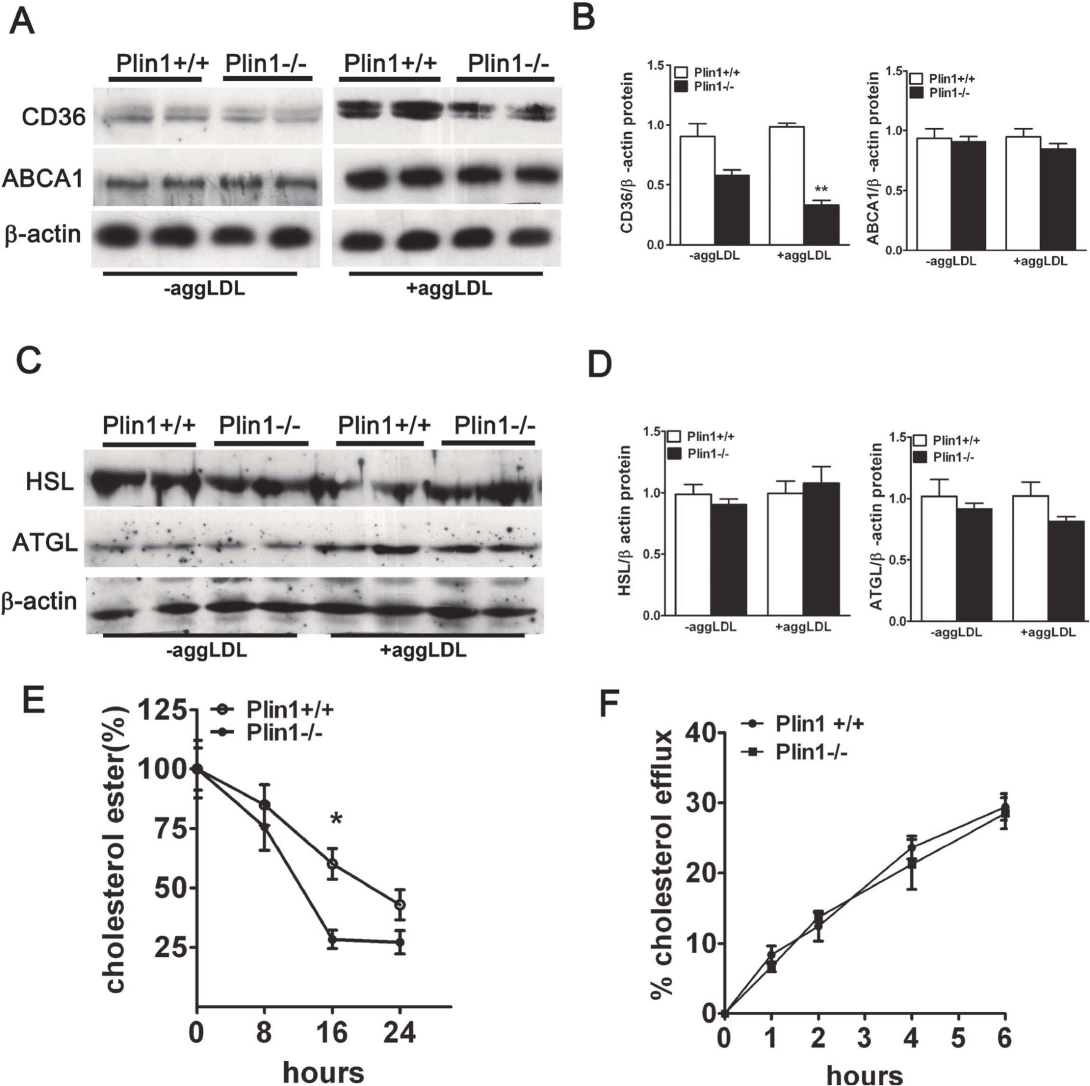
The cholesterol metabolism in macrophages. (A) Immunoblotting of CD36 and ABCA1 in peritoneal macrophages and foam cells and (B) The density of the protein band was quantitated. (C) Immunoblotting of HSL and ATGL in peritoneal macrophages and foam cells and (D) the density of the protein bands was quantitated. (E) The rate of cholesterol ester hydrolysis in peritoneal macrophages from Plin1+/+ and Plin1**-/-** mice. (F) Time-course of cholesterol efflux to ApoA-I in peritoneal macrophages from Plin1+/+ and Plin1**-/-** mice. * *p* < 0.05, ** *p* < 0.01 for Plin-/- vs. Plin+/+.

The lipid homeostasis in macrophages depends on the balance of influx and efflux of lipid in which receptors are critically involved. To explore the mechanism underlying the protective effect of Plin1 deficiency, we examined the expression of several key factors in atherogenesis in macrophages. Immunoblotting results showed that the protein level of CD36 was significantly reduced in Plin1**-/-** macrophages but the expression of ABCA1 was not altered (Fig 5A and 5B). CE deposited in macrophages was hydrolyzed by neutral cholesterol ester hydrolase, mainly by hormone-sensitive lipase (HSL) and adipose triglyceride lipase (ATGL). However, immunoblotting showed that protein expression of HSL and ATGL was not altered between the two groups (Fig 5C and 5D).

AggLDL was incubated with macrophages for 48 hours to transform them into foam cells. The CE deposited in macrophages would be hydrolysed gradually. Although macrophages from Plin1+/+ mice had more CE than macrophages from Plin1**-/-** mice, the hydrolysis of CE was faster in Plin1**-/-** macrophages (Fig 5E). Neither HSL nor ATGL expression was altered by AggLDL treatment, in macrophages from Plin-/- and wild-type mice (Fig 5C and 5D). Because the free cholesterol hydrolyzed from CE is transported by ATP-binding cassette A1 (ABCA1), then we used NBD-cholesterol to determine the cholesterol efflux rate which reflects the activity of ABCA1. We found cholesterol efflux between two groups was similar (Fig 5F). These results demonstrated that Plin1 deficiency resulted in reduced CD36 expression and faster hydrolysis of CE in Plin1**-/-** macrophages, which then restricted the foam cell formation and retarded the development of As in ApoE**-/-** background.

After lipoproteins are taken up by macrophages, CEs are hydrolyzed by cellular lipases to release free cholesterols that are effluxed to extracellular acceptors, or transported to the endoplasmic reticulum and are re-esterified to CEs by acyl-CoA: cholesterol acyltransferase-1 (ACAT1) and stored in cytoplasmic LDs [25]. The process of intracellular cholesterol homeostasis is regulated by many other molecules, such as SR-A, CD36, ABCA1, ABCG1, SR-BI, Nieman-Pick type C1 protein (NPC1), ACAT1, HSL and ATGL. There was no difference in the expression levels of any of these genes between the macrophages from Plin1+/+ mice and Plin1**-/-** mice (Data not shown). Also, quantitative PCR showed that there was no difference between the expressions of these genes in the aortic tissue of ApoE**-/-** and Plin1**-/-**ApoE**-/-** mice (Data not shown).

### **The expression of other genes of PAT family in Plin1**-/- **mice**

**Fig 6:**
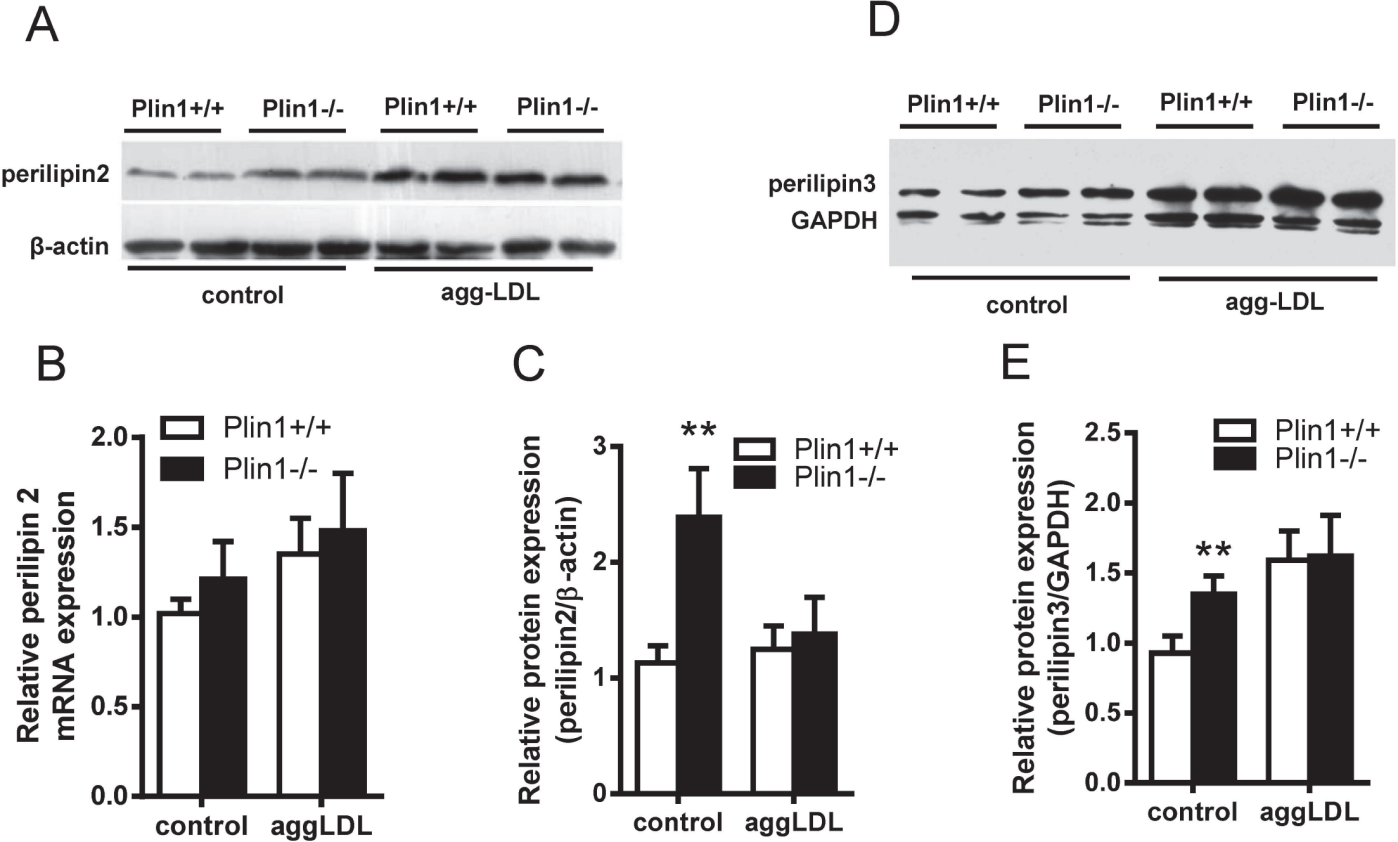
The expressions of Plin2 and Plin3 in Plin1+/+ and Plin1-/- macrophages and foam cells. (A) The expression of Plin2 protein and (B) Plin2 mRNA in the macrophages and foam cells of Plin1+/+ and Plin1**-/-** mice. (C) Protein band density of Plin2 shown in panel A. (D) Immunoblotting of Plin3 from the macrophages and foam cells of Plin1+/+ and Plin1**-/-** mice. (E) Protein band density of Plin3 shown in panel D. ** *p* < 0.01 for Plin-/- vs. Plin+/+.

Plin2, another lipid droplet protein of Plin family, was associated with As [26]. Immunoblotting showed that Plin2 was up-regulated in Plin1**-/-** macrophages (Fig 6A); this phenomenon was consistent with the compensatory increase of Plin2 in adipocytes of Plin1**-/-** mice [17]. However, the upregulation of Plin2 ceased when Plin1**-/-** macrophages were incubated with modified LDL (Fig 6A and 6C). We examined the expression of Plin2 mRNA by quantitative PCR in these macrophages and the results showed no change in Plin2 mRNA expression (Fig 6B). Similarly, Plin3 was also expressed in the mouse peritoneal microphages and was not inducible by the modified LDL in Plin1-/- microphages (Fig 6D and 6E).

## Discussion

Based on the relationship between Plin1 expression and foam cell formation we tested the hypothesis that Plin1 deficiency would possibly reduce As, by using different As-prone mouse models in absence of Plin1 gene. Although plasma levels of cholesterol and triglyceride remained unchanged between Plin1**-/-**ApoE**-/-** mice and ApoE**-/-** mice, the atherosclerotic lesions at aortic root in Plin1**-/-**ApoE**-/-** mice reduced 39% in female and 15% in male, compared to that in ApoE**-/-** mice. This is likely due to the gender difference for the earlier development of atherosclerotic plaques in female ApoE**-/-** mice [27]. The presence of Plin1 in macrophages and its function in inhibiting TG hydrolysis in adipocytes would imply that Plin1 could increase foam cell formation via suppression of CE hydrolysis. In order to define the macrophage specific role in atherogenesis, we did bone marrow transplant using marrows from Plin1+/+ and Plin1**-/-** mice as donors in another type of As-prone model, LDLR**-/-** mice. The mice receiving bone marrow cells from Plin1**-/-** mice had 49% reduction of lesion area, consistent with Pln1-/-ApoE-/- mice. This finding implicated that the deletion of Plin1 in macrophages would be responsible for decreased atherosclerotic lesions in Plin1-/- mice.

In macrophages lipoprotein derived CEs are hydrolyzed and released FCs that are re-esterified by ACAT1. The produced CEs are stored in cytoplasmic lipid droplets and are further hydrolyzed by neutral cholesterol ester hydrolyase to release FC for efflux by ABCA1 or ABCG1. This is described as cholesterol ester cycle [25]. Modified LDL was taken up mainly by CD36 and SR-A [28]. We observed that the expression of CD36 protein but not its mRNA was decreased significantly in Plin1**-/-** macrophages. It is known that CD36 deficiency markedly reduces the lipid accumulation in macrophages [29]. Hence, down-regulation of CD36 in Plin1**-/-** macrophages would be accounted for one of factors affecting the cellular CE accumulation. However, it is unclear CD36 affect lipoprotein binding or uptake, or both. This will be clarified in the future. In adipocytes Plin1 coats the lipid droplet surface as a barrier to protect the hydrolysis of the droplet lipids by lipases, but Plin1 ablation leads to the loss of this protective barrier and thus results in an elevated level of basal lipolysis [14, 17]. Our in vitro experiments showed that CEs in Plin1**-/-** macrophages disappeared faster than wild-type macrophages. The hydrolysis of CE was catalyzed by neutral cholesterol ester hydrolase (CEH) activity, which was the limiting step in cellular cholesterol efflux[30]. Based on the observations that 1) cAMP enhanced FC efflux from macrophages; 2) hormone sensitive lipase (HSL) was activated by cAMP and 3) CE was one of HSL preferred substrates, HSL was thought to be the likely candidate CEH for macrophage CE hydrolysis[31]. HSL expresses in murine macrophages and indeed provides the main CEH activity in murine macrophages [23]. Considering the role of HSL in lipolysis of adipocytes, we presume the similar mechanism in macrophages like in adipocytes, i.e., loss of Plin1 would expose accumulated CE to HSL hydrolysis. Although the expressions of mRNA and protein of HSL and ATGL were not altered, an increase in CE hydrolysis in Plin1**-/-** macrophages could be possible because the loss of Plin1 certainly abolishes the protective role of Plin1 on the droplet lipids, thus enhancing the enzymatic activity of these lipases.

Our results were in agreement with another study in which Paul found a protein related to Plin1 structurally and functionally, Plin2/ADRP, behaved similarly in its role to atherogenesis. Targeted deletion of Plin2 resulted in 40–50% reduction of aortic atherosclerotic lesions in ApoE null mice [26]. In further bone marrow transplant experiment these authors demonstrated that the lack of Plin2 in macrophages was responsible for reduced atherogenesis in Plin2 deficient mice. In their study they claimed inability to detect the signals of Plin1 in macrophages from both wild-type and Plin2**-/-** mice by Western blot. Therefore, there was no further experimental pursuit to the potential role of Plin1 in their study. We believe the discrepancy in detection of Plin1 between their data and our results was due to the anti-mouse Plin1 antibodies chosen because we demonstrated clearly the presence of Plin1 atherosclerotic lesions and localized within plaque macrophages of ApoE**-/-** mice. During adipocyte differentiation Plin2 appeared early but then disappeared after the onset of Plin1, since Plin2 was replaced by Plin1 in fully differentiating adipocytes [32]. This phenomenon prompted us to think it could also happen in transformation of macrophages to foam cells. Our immunostaining and immunoblotting data showed that both Plin1 and Plin2 proteins were expressed in the atherosclerotic plaques of ApoE-/- mice, and in some area Plin1 was colocalized with CD68, in the atherosclerotic microphages. Plin2 is a lipid-droplet marker ubiquitously expressed in all types of cells except for mature adipocytes [11]. Plin2, like Plin3 and Plin4, has been long assumed to prevent the LDs from the hydrolysis by cellular lipase, but this putative role remains not to be accessed. By contrast, the functional roles of Plin1 in both LD storage and degradation have been firmly established. Native Plin1 proteins barrier and protect the LDs from access of HSL and ATGL, thus prevent LD degradation and enhance LD formation [10, 11]. On catecholamine stimulation, Plin1 is phosphorylated by protein kinase A and then induces the translocation of HSL from the cytosol to the LD surface and also indirectly activated ATGL [10, 11]. In human atherosclerotic plaques, Plin1 protein is highly expressed in unstable atherosclerotic plaques and also expressed considerably in stable plaques [12]. Plin1 is mainly assembled in 3 subcellular locations of human atherosclerotic plaques, cells surrounding some cholesterol clefts, foam cells, and endothelial cells in newly formed vessels [12]. This observation is consistent with our present finding of Plin1 expression pattern in atherosclerotic plaques of ApoE-/- mice. In light of our immunostaining results, the circular fluorescence signals of Plin1 and CD68 indicated that Plin1 was associated with the LDs in CD68-labeled foam cells, but the irregular signal patterns implicated that Plin1 was also assembled in irregular vessel endothelium and in the cells sounded with cholesterol crystals. Given the fundamental role of Plin1 in protecting LD lipids from lipase hydrolysis, it is reasonably speculated that the assembly of Plin1 might function to reduce lipolysis and hence increase lipid retention in plaques. This could be a cellular basis by which Plin1 deficiency in bone marrow-derived cells resulted in reduced atherosclerotic lesions in mice.

Our results were contradictory to the report by Langlois et al.[15], in which they found Plin1 deficiency in LDLR null mice increased As lesion development and interpreted as the consequence of 7 fold up-regulation of scavenger receptor A. There was no explanation by the authors on how Plin1 deficiency could lead to such tremendous upregulation of SR-A. Though we could not fully understand what caused the discrepancy between our and their results, we did find some disparate points: 1). We quantitated the As lesions both in the aortic root by multiple consecutive cryosections under microscope and in the en face entire aorta by Oil red staining,while they only did whole aorta en face lesion assessment by Sudan IV staining. 2).We chooses both males and females for the study and found significant difference in atherogenic lesions only in females after HFD feeding for 12 weeks. In their study they just had males and found increased As lesions after 20 weeks HFD feeding but not at 10 weeks interval. 3).We found consistently reduced As lesions in either ApoE null background when whole body Plin1 was deleted, or in LDLR null female mice when Plin1 in macrophages was depleted by bone marrow transplant approach. In contrast, they only had whole body Plin1 deficiency on LDLR null background. In explanation of the 7 fold up-regulation of SR-A expression as the cause for increased As lesions in Plin1 deficiency, they also showed a striking increase of mRNAs of ABCA1 and ABCG1 for nearly 20 and 5 folds, respectively. Though these changes were interpreted as response to the up-regulated SR-A in Plin1 null mice,such robust increase of ABCA1 and ABCG1 would certainly diminish As lesions in theory via resulting super Cholesterol efflux. For instance, ABCA1 transgenic expression in mice resulted in 3 to 5 fold increase of ABCA1 in aorta or in macrophage and such increase in ABCA1 led to markedly reduction of aortic lesion areas in As-prone ApoE or LDLR null background [33]. Therefore, the inconsistent relationship in upregulated SR-A, ABCA1, ABCG1 and Plin1 deficiency with enhanced As lesions would require further careful examination.

Taken together, Plin1 deficiency in macrophages decreased lipid deposits by inhibition of foam cell formation and hence reduced As lesions in arterial walls. We postulated the major cause for reduced lipid accumulation in Plin1**-/-** macrophages was due to the disappearance of the barrier composed by Plin1, which facilitates the interactions of lipases with lipid droplets and results in faster hydrolysis of LD. In addition to this macrophage LD hydrolysis, down-regulation of CD36 could lead less uptake of modified LDL, which may also to some extent contribute to reduced foam cell formation.
